# Dopamine and Citicoline-Co-Loaded Solid Lipid Nanoparticles as Multifunctional Nanomedicines for Parkinson’s Disease Treatment by Intranasal Administration

**DOI:** 10.3390/pharmaceutics16081048

**Published:** 2024-08-07

**Authors:** Stefano Castellani, Giorgia Natalia Iaconisi, Francesca Tripaldi, Vito Porcelli, Adriana Trapani, Eugenia Messina, Lorenzo Guerra, Cinzia Di Franco, Giuseppe Maruccio, Anna Grazia Monteduro, Filomena Corbo, Sante Di Gioia, Giuseppe Trapani, Massimo Conese

**Affiliations:** 1 Department of Precision and Regenerative Medicine and Ionian Area (DiMePRe-J), University of Bari “Aldo Moro”, 70125 Bari, Italy; stefano.castellani@uniba.it; 2 Department of Biological and Environmental Sciences and Technologies, University of Salento, 73100 Lecce, Italy; giorgianatalia.iaconisi@unisalento.it; 3 Department of Pharmacy-Drug Sciences, University of Bari “Aldo Moro”, 70125 Bari, Italy; francesca.tripaldi97@gmail.com (F.T.); filomena.corbo@uniba.it (F.C.); giuseppe.trapani080@uniba.it (G.T.); 4 Department of Biosciences, Biotechnologies and Environment, University of Bari “Aldo Moro”, 70125 Bari, Italy; vito.porcelli@uniba.it (V.P.); eugenia.messina@uniba.it (E.M.); lorenzo.guerra1@uniba.it (L.G.); 5 CNR-IFN Bari, Bari Via Amendola 173, 70126 Bari, Italy; cinzia.difranco@cnr.it; 6 Omnics Research Group, Department of Mathematics and Physics “Ennio De Giorgi”, University of Salento and INFN Sezione di Lecce, Via per Monteroni, 73100 Lecce, Italyannagrazia.monteduro@unisalento.it (A.G.M.); 7 CNR-NANOTEC Institute of Nanotechnology, Via per Monteroni, 73100 Lecce, Italy; 8 Department of Clinical and Experimental Medicine, University of Foggia, 71122 Foggia, Italy; sante.digioia@unifg.it (S.D.G.); massimo.conese@unifg.it (M.C.)

**Keywords:** citicoline, dopamine, RPMI 2650 cells, SH-SY5Y cells, OxyBlot assay

## Abstract

This work aimed to evaluate the potential of the nanosystems constituted by dopamine (DA) and the antioxidant Citicoline (CIT) co-loaded in solid lipid nanoparticles (SLNs) for intranasal administration in the treatment of Parkinson disease (PD). Such nanosystems, denoted as DA-CIT-SLNs, were designed according to the concept of multifunctional nanomedicine where multiple biological roles are combined into a single nanocarrier and prepared by the melt emulsification method employing the self-emulsifying Gelucire^®^ 50/13 as lipid matrix. The resulting DA-CIT-SLNs were characterized regarding particle size, surface charge, encapsulation efficiency, morphology, and physical stability. Differential scanning calorimetry, FT-IR, and X ray diffraction studies were carried out to gain information on solid-state features, and in vitro release tests in simulated nasal fluid (SNF) were performed. Monitoring the particle size at two temperatures (4 °C and 37 °C), the size enlargement observed over the time at 37 °C was lower than that observed at 4 °C, even though at higher temperature, color changes occurred, indicative of possible neurotransmitter decomposition. Solid-state studies indicated a reduction in the crystallinity when DA and CIT are co-encapsulated in DA-CIT-SLNs. Interestingly, in vitro release studies in SNF indicated a sustained release of DA. Furthermore, DA-CIT SLNs displayed high cytocompatibility with both human nasal RPMI 2650 and neuronal SH-SY5Y cells. Furthermore, OxyBlot assay demonstrated considerable potential to assess the protective effect of antioxidant agents against oxidative cellular damage. Thus, such protective effect was shown by DA-CIT-SLNs, which constitute a promising formulation for PD application.

## 1. Introduction

Among the neuroactive substances which, nowadays, are attracting much attention for their feasible clinical application in the treatment of neurological disorders, citicoline (Cytidine 5′-Diphosphocholine or cytidine diphosphate choline, CIT) merits consideration for its several benefits. CIT, indeed, is an important intermediate in the biosynthesis of cell membrane phospholipids and its main activity is connected to the improvement of the human psychomotor vigilance, arousal, and visual work [1]. CIT’s capability in re-establishing normal brain and cerebral cortex structure has also been confirmed in several models of neurological disorders, including its beneficial outcome to block the simvastatin unwanted side effects in patients suffering from Alzheimer’s disease (AD) [2,3]. From a clinical viewpoint, CIT adjuvant therapy has also shown favorable effects in Parkinson’s disease (PD), as shown in a recent comprehensive review article on this topic [4].

As a consequence, in the last couple of decades, several research efforts have been devoted to formulate CIT in nanosized lipid-based pharmaceutical dosage forms in order to bypass the obstacle represented by the blood–brain barrier (BBB) and to reach the central nervous system (CNS). Thus, for the treatment of cerebral ischemia, stealth immunoliposomes encapsulating CIT were evaluated for their diagnostic and therapeutic properties in vivo, in an animal model of cerebral ischemia [5]. Based on the abovementioned arguments, it appears that CIT may play a key role in the treatment of neurological diseases. In this regard, it is important to remember that, currently, with an ever-increasing aging population worldwide, the incidence of neurological disorders is continuously growing, resulting in over one in three people affected by neurological conditions, the leading cause of illness and disability worldwide [6]. However, in spite of the huge efforts made by the scientific community, there is still a need to develop new and more effective disease-modifying strategies to hinder this increasing incidence of neurological disorders. This worrying incidence is mainly explained by a delivery problem, namely, the inability of most potentially active-at-CNS-level substances to overcome the BBB in therapeutic amounts.

In this regard, in the last years, most interest has been focused on the administration of drugs by intranasal route since it may offer a noninvasive bypass to the BBB. The intranasal delivery offers well-established advantages over the oral administration which, on the other hand, is characterized by a variety of formulation options and better patient compliance [7,8]. The advantages of the nasal administration over the oral one include the avoidance of first-pass metabolism and rapid onset of action [9]. In addition, one of the most promising applications of nasal drug delivery is the nose-to-brain delivery, which allows a variety of neuroactive agents to be conveyed directly to the brain in therapeutic amounts, exploiting the fact that the olfactory mucosa is the region of nasal cavity not protected by the blood–brain barrier [10,11].

In the last decades, nanodelivery systems have been widely used for the targeting of brain diseases, including polymeric and inorganic nanoparticles of small dimensions such as gold and other metallic nanoparticles prepared by laser ablation, due to their ability to increase drug bioavailability and BBB crossing [12,13,14]. Notably, lipid nanosystems constituted by hydrophobic and amphiphilic molecules are also able to form different structures such as vesicles and micelles in aqueous medium, and possess a great potential for nose-to-brain delivery application [12]. Such lipid-based nanosystems comprise solid lipid nanoparticles (SLNs), nanostructured lipid carriers, and liposomes, among others.

A further innovative concept which, in recent years has been introduced for the treatment of brain diseases, is denoted as “multifunctional nanomedicines”, consisting of the use of nanocarriers able to encompass multiple disease fronts through the co-delivery of two or more active substances to the target site [15]. Thus, for instance, it is well known that oxidative stress (i.e., the imbalance between the biochemical production pathways of the reactive oxygen species (ROS) and the cellular antioxidant cascade resulting in molecular damage) and mitochondrial dysfunction play a crucial role in the pathogenesis of neurodegenerative diseases, including AD and PD [16,17,18]. It is in agreement with the experimental observation that, in the PD patients’ brain, the presence of high levels of ROS occurs, leading to DA neuron loss, which constitutes the hallmark typical of PD [19]. Consequently, it is suitable to evaluate for brain delivery, by intranasal administration, the combination of an anti-Alzheimer’s or a dopaminergic drug and an antioxidant agent co-encapsulated in a lipid nanocarrier as multifunctional nanomedicine for the treatment of AD or PD, respectively [20].

The advantages of these multifunctional nanomedicines over the monofunctional ones in brain delivery include enhanced bioavailability, targeted delivery, and controlled drug release [15]. As for the advantages of these multifunctional nanomedicines over other nanomedicines designed specifically for PD, they may be clearly demonstrated by the reported co-delivery of curcumin and piperine in lipid-based nanocarriers to an animal model of PD [15]. It was shown that such combination led to reduced levels of oxidative stress and cell apoptosis due to the former therapeutic agent, while piperine was able to prevent the formation of oligomers of alpha-synuclein proteins, unlike the untreated control [15]. An additional advantage of these multifunctional nanomedicines with high relevance in PD treatment is the protection of the cargo against unfavorable environments. Thus, catecholamines such as DA can be appropriately protected regarding the spontaneous autoxidation reaction under neutral/alkaline conditions to give cytotoxic products by the simultaneous presence of an antioxidant agent in the same nanosystem.

In this context, we recently reported on the evaluation of the neurotransmitter dopamine (DA) and the antioxidant grape-seed-derived proanthocyanidins (grape seed extract, GSE) co-loaded in solid lipid nanoparticles (SLNs) to achieve a more favorable PD treatment through nose-to-brain delivery [21]. By flow cytometry, we found a higher SLN internalization both in olfactory ensheathing cells and in neuronal SH-SY5Y cells when GSE was co-encapsulated, rather than adsorbed onto the particles. In addition, Franz diffusion cell experiments demonstrated a higher permeation of DA from both SLN types through the porcine nasal mucosa compared to unencapsulated DA [21].

As part of our ongoing research program aimed at the screening of some combinations of neurotransmitter DA and antioxidant agent co-loaded in SLNs for a more effective PD treatment through nose-to-brain delivery, we report herein on the technological and biological characterization of the nanosized platform denoted as DA-CIT-SLNs, where the neurotransmitter DA and the antioxidant CIT are co-loaded in SLNs. It should be noted that we previously investigated the potential of SLNs for CIT-alone vectorization [22], selecting such nanocarriers for their promising features such as safety and stability. Moreover, they can be prepared on a large scale and proposed to patients following several delivery routes. In particular, SLNs are extensively used to improve the formulation of hydrophobic drugs, but, relevantly, selected formulation protocols also allow hydrophilic drug substances to be encapsulated in these nanocarriers [23]. In this regard, we developed a preparative method of SLNs, enabling the encapsulation of both lipophilic and hydrophilic drugs, consisting of the melt emulsification method employing Gelucire^®^ 50/13 (a self-emulsifying mixture of PEG-esters (stearoyl polyoxyl-32 glycerides), a small glyceride fraction and free PEG chains) as lipid matrix [24,25,26]. However, our objective to co-encapsulate two substances as DA and CIT in the same lipid matrix is not a simple task; it is actually particularly challenging due to their hydrophilic character (calculated log Po/w = −1 and −4, respectively (computed by XLogP3 3.0, PubChem release 2021.10.14).

Following the mentioned preparative method, we successfully formulated Gelucire^®^ 50/13-based DA-CIT-SLNs which are herein characterized for their physicochemical properties including particle size, surface charge, and encapsulation efficiency. In addition, we carried out the characterization of these SLNs at solid state using differential scanning calorimetry (DSC) and X ray diffraction (XRD), and in vitro release and physical stability studies were also performed. From a biological viewpoint, cytobiocompatibility was ascertained in the presence of human nasal RPMI 2650 and neuronal SHSY-5Y cells, by working with two different DA concentrations. Then, OxyBlot assay performed in the neuronal SHSY-5Y cells also allowed us to screen different SLN formulations, pointing out the protective role exerted by the antioxidant CIT, and after nanoencapsulation in SLNs.

Last but not least, it should be also noted that the DA-CIT-SLNs approach, proposed herein as a new option of PD treatment, in a broader perspective, may be also adapted in designing appropriate combinations of drug/antioxidant agents to manage further neurological disorders by intranasal administration, in addition to PD.

## 2. Materials and Methods

### 2.1. Materials

Gelucire^®^ 50/13 was received as a gift by Gattefossè (Milan, Italy). Citicoline sodium salt (CIT) was kindly provided as a gift from Esseti Farmaceutici s.r.l. (Pomezia, Italy). Polysorbate 85 (Tween^®^ 85), acetic acid (AcOH), KBr, porcine stomach mucin (type II, sialic acid ~1%), dopamine hydrochloride, carboxyl ester hydrolase (E.C. 3.1.1.1, 15 units/mg powder), and 2,2-diphenyl-1-picrylhydrazyl (DPPH) were purchased from Sigma Aldrich (Milan, Italy). Dialysis tubes with an MWCO 3.5-4 kDa were purchased from Spectra Labs (Milan, Italy). Advanced Minimum Essential medium (A-MEM) was purchased from Gibco-Thermo Fisher Scientific (Waltham, MA, USA). Heat-inactivated fetal bovine serum (FBS) was purchased from Euroclone S.p.A (Pero, Italy). GutaMAX™ supplement was acquired from Biowest (Nuaillé, France). Trypsin EDTA 0.25% was purchased from Elabscience (Huston, TX, USA). Alamar Blue and Resazurin were purchased from Bio-Rad (Hercules, CA, USA) and Biotium (Fremont, CA, USA), respectively. In this work, double-distilled water was employed, and all other chemicals were of reagent grade.

### 2.2. Preparation of DA-CIT-SLNs-60

DA-CIT-SLNs were prepared following the melt emulsification method previously reported by us [24,25,26]. Briefly, 120 or 60 mg of Gelucire^®^ 50/13 were melted at 70 °C, and in a separate vial, 1.37 mL of a diluted AcOH solution (0.01%, *w*/*v*) containing the surfactant (Tween 85^®^, 60 mg) was heated at 70 °C. Then, 10 mg of each active principle CIT and DA were poured in the AcOH solution before the addition of the resulting mixture to the melted lipid at 70 °C. By homogenization of this mixture at 12,300 rpm for 3 min with an UltraTurrax model T25 apparatus (Janke and Kunkel, IKA^®^-Werke GmbH & Co., Staufen, Germany), an emulsion was obtained. By cooling at room temperature of the nanosuspension, the resulting SLNs were subjected to centrifugation (Eppendorf 5415D, Hamburg, Germany) at 13,200× *g*, 45 min, and the obtained pellet was used for successive studies while the supernatant was discarded. Throughout the study, based on the Gelucire^®^ 50/13 amount employed, SLNs were denoted as “DA-CIT-SLNs-60” and “DA-CIT-SLNs-120”.

### 2.3. Quantification of DA and CIT

The DA and CIT quantifications were performed by HPLC as follows. HPLC equipment consisted of a Waters Model 600 pump (Waters Corp., Milford, MA, USA), a Waters 2996 photodiode array detector, and a 20 μL loop injection autosampler (Waters 717 plus). A Synergy Hydro-RP (25 cm × 4.6 mm, 4 μm particles; Phenomenex, Torrance, CA, USA) was the stationary phase, and 0.02 M potassium phosphate buffer, pH 2.8: CH_3_OH 90:10 (*v*:*v*), was employed as mobile phase. The isocratic mode was selected for column elution at the flow rate of 0.7 mL/min at the wavelength of 280 nm and, under such chromatographic conditions, the retention times of CIT and DA were 3.5 min and 6.5 min, respectively. Under the previous mentioned HPLC conditions, the quantifications of CIT and DA were performed in the ranges 80 μg/mL–24 ng/mL and 600–2 μg/mL, respectively.

For DA and CIT content evaluation in SLNs, after lyophilization, particles underwent enzymatic digestion by esterases [27]. In particular, 1−2 mg of freeze-dried SLNs and 1 mL of a solution of such enzyme at 12 I.U./mL in phosphate buffer (pH 5) were incubated for 30 min in an agitated (40 rpm/min) water bath at 37 °C (Julabo, Milan, Italy). Then, the resulting mixture was centrifuged (16,000× *g*, 45 min, Eppendorf 5415D) and the supernatant was analyzed by HPLC for DA and CIT quantitative determination, as reported above.

The encapsulation efficiency (E.E.%) was calculated using Equation (1):

E.E.% = DA (or CIT) in the supernatant after esterase assay/Total DA (or CIT) × 100 (1)

where total DA (or CIT) is intended as the starting amount of each substance used for SLN preparation. This study was performed in triplicate.

### 2.4. Physicochemical Characterization of SLNs

For all SLNs, particle size and polydispersity index (PDI) determinations at 25 °C took place after dilution in double-distilled water (1:1, *v*:*v*) in disposable polystyrene latex cuvettes. A Zetasizer NanoZS (ZEN 3600, Malvern, UK) apparatus was used following photon correlation spectroscopy (PCS) mode with angle detection set at 90°. Via the use of laser Doppler anemometry (Zetasizer NanoZS, ZEN 3600, Malvern, UK) following dilution 1:20 (*v*:*v*) in the presence of KCl (1 mM, pH 7), zeta potential measurements were also carried out at 25 °C in disposable polystyrene latex folded cuvettes. The particle size, PDI, and zeta potential values were each measured in eight sample replicates [28]. Transmission electron microscopy (TEM, FEI Tecnai 12 TEM, Eindhoven, The Netherlands), equipped with a LaB6 filament operating at 120 kV, was adopted to investigate the nanoparticle morphology in the dried state. For SLN observations, drops of suspensions were deposited on Formvar^®^-coated Cu grid (300 mesh, Agar Scientific, Stansted, Essex, UK). The microscope was calibrated using the S106 Cross Grating (2160 lines/mm, 3.05 mm) supplied by Agar Scientific. Corrections concerning alignments and astigmatism were carried out following factory settings and fast Fourier transform processing, respectively.

### 2.5. Solid-State Studies

#### 2.5.1. Fourier Transform Infrared (FT-IR) Spectroscopy

FT-IR spectra were acquired in KBr discs using 2–5 mg of pure DA, CIT, CIT-SLNs, and lyophilized DA-CIT-SLNs-60 (72 h of a freeze-drying cycle, Lio Pascal 5P, Milan, Italy). A Perkin Elmer 1600 FT-IR spectrometer (Perkin Elmer, Milan, Italy) processed all the spectra (r.t., 4000–400 cm^−1^ wavenumber range), showing a resolution of 1 cm^−1^.

#### 2.5.2. Differential Scanning Calorimetry (DSC)

DSC calorimetric runs were carried out for bulk materials such as pure DA and CIT, freeze-dried CIT-SLNs, and DA-CIT-SLNs-60, using a Mettler Toledo DSC 822e STARe was 202 System combined with DSC MettlerSTARe Software STARe SW V6.0 (Mettler Toledo, Milan, Italy). Freeze-dried particles (5 mg) of each product were placed in an aluminum pan and hermetically sealed. The scanning rate was of 5 °C/min under a nitrogen flow of 20 cm^3^/min, and the temperature range was set from 25 to 275 °C for all samples. Following the procedure of the MettlerSTARe Software, the DSC apparatus was calibrated using indium (99.9%). Each thermal run was replicated three times.

#### 2.5.3. X-ray Powder Diffraction (XRPD)

X-ray diffraction patterns of pure DA, freeze-dried plain SLNs, and DA-CIT-SLNs-60 were acquired using an X’Pert PRO (PANalytical, Malvern, UK) system. For safety of comparison, free CIT and CIT-SLNs X-ray diffraction patterns were acquired as described in [22]. The data were collected at room temperature in the 2θ range of 10°–50°, with a step size of 0.02°.

### 2.6. Physical Stability of DA-CIT-SLNs

The physical stability of DA-CIT SLNs-60 dispersions was monitored by particle size measurements during storage at 4 °C in the refrigerator up to 1 week and at 37 °C up to 24 h as well. Precisely, a water bath (Julabo, Milan, Italy) set at the temperature equal to 37 °C with agitation of 40 rpm/min was adopted for SLN incubation. At different time points, particle size was checked, and cuvette preparation followed the approach described in Section 2.4. Physical stability studies were carried out in triplicate at each temperature.

### 2.7. In Vitro Release Tests

To start in vitro release studies, freeze-dried DA-CIT SLNs-60 (corresponding to 1–1.2 mg of both DA and CIT) was dispersed in 1.5 mL of double-distilled water in a dialysis bag soaked in the receiving medium thermostatted at 37 ± 0.1 °C in an agitated (40 rpm/min) water bath (Julabo, Milan, Italy). The receiving medium was constituted of 40 mL of simulated nasal fluid (SNF) mixed with 0.25% (*w*/*v*) of mucin (pH of the mixture was equal to 6.0) [29]. The release study was conducted for 24 h, and at scheduled time points, 0.8 mL of the receiving medium were withdrawn and replaced with 0.8 mL of fresh medium. Then, centrifugation took place at 16,000× *g* for 45 min (Eppendorf 5415D, Germany), and the amounts of DA (or CIT) delivered were determined in the resulting supernatants obtained after centrifugation. The release experiments in SNF containing mucin were performed in triplicate.

### 2.8. Cytotoxicity Assessment in RPMI 2650 and in SH-SY5Y Cell Model Lines

Neuronal SH-SY5Y cells, grown as previously described [30], and RPMI 2650, grown as previously described [26], were plated in a 96-plastic culture plate (BD, Franklin Lakes, NJ, USA) for 24 h at a density of 40,000 cells per well. Then, for both cell lines, the culture medium was replaced with 200 μL of fresh medium containing different dilutions of free DA, free CIT, DA-SLNs, CIT-SLNs, DA-CIT-SLNs-60, and plain SLNs. In a set of experiments, DA was used at 100 μM and CIT at 23 μM, while in another set of experiments, DA was used at 50 μM and CIT at 12.5 μM. Volumes of plain SLNs were chosen to obtain the same lipid amounts of the other preparations. In these conditions, cells were grown at 37 °C for 24 h. The 0.1% Triton-X100-treated cells were used as a positive control. Then, after removing the medium and washing two times with PBS, viability was evaluated by resazurin-based colorimetric assay according to the manufacturer’s protocol (Biotium, Fremont, CA, USA) [31,32,33].

Briefly, the fluorescence of solubilized resorufin was measured at the emission wavelength of 590 nm with the excitation wavelength at 544 nm by using a microplate reader FLUOstar Omega microplate reader (BMG Labtech, Ortenberg, Germany). Then, 0.1% Triton X-100 treatment was used as a positive control, and each experiment was performed three times.

### 2.9. Permeation Studies across RPMI-2650 Cell Monolayer

To evaluate the transport capability of DA, CIT, SLN CIT, and DA-CIT SLNs through a cellular monolayer, RPMI 2650 cells were grown at an air–liquid interface onto Transwell semipermeable filters in order to acquire morphological and functional features resembling a polarized barrier epithelium. Briefly, cells were seeded (3 × 10^5^ cells/cm^2^) on permeable inserts (Transwell^®^, 0.4 μm pore size, 0.33 cm^2^ growth area; Corning, Tewsbury, MA, USA) for two days and then at an air–liquid interface for 14 days to form a fully confluent and differentiated monolayer [34,35]. Trans-epithelial electrical resistance (TEER) was evaluated as indicator of epithelium tightness using an EVOM resistance meter and STX 2 electrodes (World Precision Instruments, Sarasota, FL, USA). Blank filter values were subtracted, and the values were calculated considering the surface area of the inserts (average TEER after 14 days in air liquid interface was 80 ± 8 Ω cm^2^). Powders of DA (or CIT) or freeze-dried samples of CIT-SLNs and DA-CIT-SLNs-60 were suspended in medium without phenol red to obtain a final concentration of 0.4 mg/mL DA and 0.3 mg/mL CIT. Then, 100 μL of each sample were added to the apical side of the insert, while the basal side contained 500 μL of culture medium. Culture medium without sample was considered as negative control. After 3, 6, and 24 h of incubation, DA and/or CIT content(s) were evaluated both in the apical and basal medium, by withdrawing 0.3 mL of the receiving medium and replacing with 0.3 mL of fresh medium. Then, each sample was centrifuged at 16,000× *g* for 45 min (Eppendorf 5415D, Germany), and the amounts of DA and/or CIT delivered were quantified in the resulting supernatants by HPLC, as described in Section 2.3. Each assay was performed in triplicate.

### 2.10. DPPH Assay for In Vitro Antioxidant Activity Evaluation

The in vitro antioxidant activity of the control powders of DA and CIT, plain SLNs, CIT-SLNs, and DA-CIT-SLNs-60 was evaluated using the DPPH test with slight modifications [30,36,37]. Firstly, an ethanolic solution of DPPH was obtained at the concentration of 0.001% *w*/*v* and then diluted to 8 × 10^−4^% (*w*/*v*). After freeze-drying of the SLNs, 0.5 mL of each sample previously redispersed in ethanol was reacted with 2.5 mL of the diluted DPPH solution for 60 min at room temperature in the dark, and for each sample, the corresponding absorbance was recorded at the wavelength of 514 nm via the use of a Perkin-Elmer Lambda Bio 20 spectrophotometer (Milan, Italy). Blanks were obtained by filling the cuvettes with 0.5 mL of ethanol and 2.5 mL of the 8 × 10^−4^% (*w*/*v*) solution in DPPH. The lipid Gelucire^®^ 50/13 was dispersed in acetone at 5 mg/mL by vortexing and, afterwards, treated as above with DPPH reactant. For all formulations under investigation, antioxidant activity (AA) was calculated using Equation (2) and expressed in percentages:

AA (%) = (1 − As/Ab) × 100 (2)

where As is the sample absorbance and Ab is the absorbance of the radical.

### 2.11. OxyBlot Assay

Carbonylated proteins, whose content is widely used as a marker for oxidative stress [38], were detected by using the OxyBlot™ Protein Oxidation Detection Kit (Merck Millipore, Billerica, MA, USA) according to the manufacturer’s instructions. Proteins obtained from each sample in equal amounts were split into two aliquots, and each one underwent a denaturation cycle by using a 6% SDS (w/v) solution. Subsequently, one aliquot was derivatized with 2,4-Dinitrophenylhydrazine (DNPH) solution, while the other aliquot was treated with derivatization-control solution, serving as a nonderivatized control. The derivatized and nonderivatized proteins were then separated using 12% SDS-PAGE and transferred onto a nitrocellulose membrane. After blocking nonspecific binding, the membrane was incubated with an antidinitrophenyl primary antibody (1:150, polyclonal; Merck Millipore, Billerica, MA, USA, 90451) dilution in Tris-buffered saline (TBS), 0.1% Tween-20, and 5% milk, at room temperature for 1 h. Following washing, the membrane underwent an additional incubation with goat antirabbit secondary antibody (Merck Millipore, Billerica, MA, USA, 90452) diluted 1:300 in TBS, 0.1% Tween-20, and 5% milk, at room temperature for 1 h. Immunoreactive protein bands were visualized using ECL chemiluminescence substrate and the ChemiDoc Imaging System (Bio-Rad, Hercules, CA, USA). The intensities of oxidized protein bands in each lane were quantified using Image Lab software (version 6.1 Bio-Rad, Hercules, CA, USA).

### 2.12. Statistics

Statistical analyses were carried out by Prism v. 5.0 (GraphPad Software Inc., La Jolla, CA, USA). Data are expressed as mean ± SD. Multiple comparisons were based on one-way analysis of variance (ANOVA). Either Bonferroni’s or Tukey’s post hoc test were carried out in all cases, except for OxyBlot assay results. The statistical analysis led to differences being considered significant when *p* < 0.05. For cell viability experiments, statistical significance was evaluated by a two-tailed unpaired Student’s t-test. Significant differences were obtained when *p* < 0.05.

## 3. Results

### 3.1. Preparation and Characterization of SLNs

**Figure 1:**
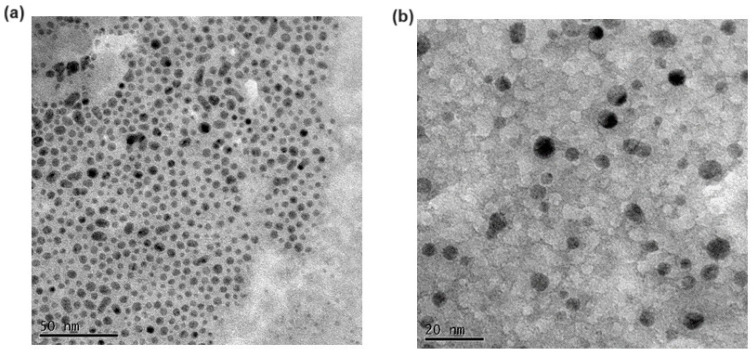
Transmission electron microscopy images of DA-CIT-SLNs-60 at the dried state. Panel (**a**) scale bar is 50 nm. Panel (**b**) scale bar is 20 nm.

Table 1 shows the main physicochemical properties of the DA-CIT SLNs herein prepared starting from two different amounts of the lipid Gelucire^®^ 50/13. A statistically significant difference compared to plain SLNs occurred in terms of particle size when the amount of the lipid Gelucire^®^ 50/13 was set at 120 mg. Indeed, DA-CIT-SLNs-120 exhibited an average diameter of 405 nm, whereas the particle size observed for the formulation DA-CIT-SLNs-60 was seen to be similar to plain SLNs and, in addition, smaller than both CIT-SLNs and DA-SLNs previously described by us [22,25] (Table 1). Moreover, the same trend was observed also for PDI values; namely, the PDI value of DA-CIT-SLNs-120 was higher than plain SLNs, suggesting a possible plurimodal size distribution. On the other hand, the PDI value of DA-CIT-SLNs-60 was comparable to that of plain SLNs, which may even imply a broad monomodal size distribution. The results reported in Figure 1 and Figure S1, which deal with TEM visualization and size distribution of both DA-CIT-SLNs herein discussed, respectively, clearly show that the size distribution of DA-CIT-SLNs-60 is of broad monomodal type, while that of DA-CIT-SLNs-120 corresponds to a bimodal (two populations) size distribution (Figure S1a,b, respectively). In addition, Figure 1 shows that both DA-CIT-SLNs were almost round-shaped nanocarriers.

For all tested DA-CIT-SLNs, zeta potential values were comparable with plain SLNs and found to be negatively charged, unlike DA-SLNs and CIT-SLNs, which were practically neutral from a surface charge point of view. This may suggest that the introduction of two hydrophilic substances (i.e., DA and CIT) slightly improves the physical stability of the corresponding nanocarriers compared to the single introduction of them. Notably, high levels of DA and CIT contents were determined by HPLC and, in detail, the E.E.% of DA were equal to 77 ± 7% and 65 ± 3%, while the E.E.% of CIT were equal to 75 ± 2% and 59 ± 8% for DA-CIT-SLNs-60 and DA-CIT-SLNs-120, respectively (Table 1). However, due to the unfavorable particle size and the lower neurotransmitter content of DA-CIT-SLNs-120, we discarded these nanocarriers and, thus, they were not subjected to further in vitro studies.

### 3.2. Physical Stability

**Figure 2:**
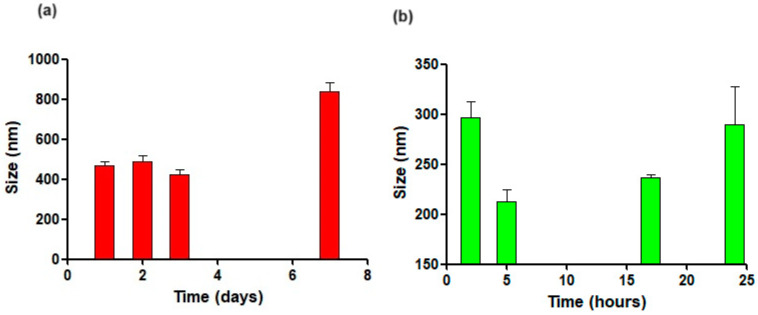
Physical stability of DA-CIT-SLNs-60 particle size over time and temperature: (**a**) 4 °C for one week; (**b**) 37 °C for 24 h. The control was the mean particle size of DA-CIT-SLNs-60 reported in Table 1.

The assessment of the physical stability of DA-CIT-SLNs-60 at 4 °C and 37 °C was carried out following the evolution of particle size of such nanocarriers after storage at mentioned temperatures over the time reported in Figure 2. As shown in Figure 2a, size enlargement observed over the time at 4 °C was relevant from one day of storage, but no color changes appeared under visual inspection up to one week. At 37 °C, particles’ mean diameters increased over time (Figure 2b), even though this enhancement was lower than that observed at 4 °C. However, after 24 h of storage at 37 °C, color changes occurred, suggesting possible neurotransmitter decomposition; hence, the study was stopped. To substantiate the hypothesis of possible neurotransmitter decomposition, the UV–Vis spectra (Agilent/HP 8453 UV–Vis Spectrophotometer-Spectroscopy System) of aliquots of DA-CIT-SLNs-60 stored at 37 °C for 24 h were examined. Such samples showed intense absorbances at the wavelengths of 450 nm, attributable to aminochrome, which is the key intermediate of the neurotransmitter autoxidation process leading to color changes, precipitate formation, or synthesis of polymeric substances (i.e., neuromelanin) [40,41].

### 3.3. Solid-State Studies

**Figure 3:**
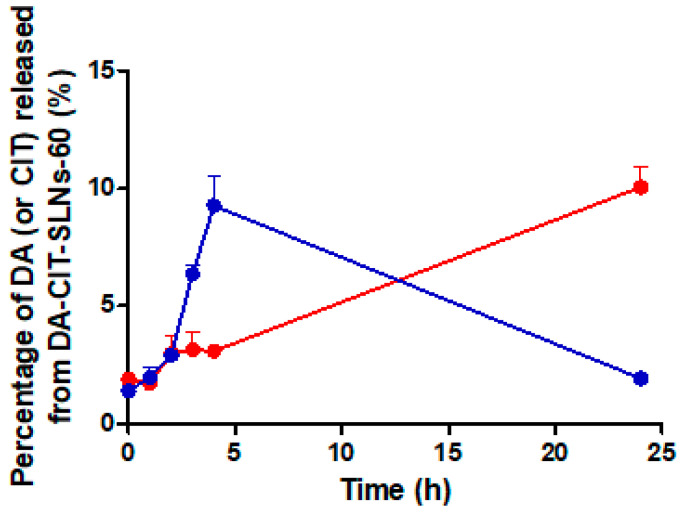
In vitro release of DA (red) and CIT (blue) from DA-CIT-SLNs-60 in SNF at 37 °C.

The solid-state characteristics of the prepared DA-CIT-SLNs-60 were assessed by spectroscopy (FT-IR), thermal analysis (DSC), and X-ray powder diffraction (XRPD) studies, and the relative results are summarized in Figure 3.

#### 3.3.1. FT-IR Spectroscopy

As already observed for the FT-IR spectrum of others Gelucire^®^ 50/13-based SLNs, DA-CIT-SLNs-60 also showed absorption bands due to Gelucire^®^ 50/13 lipid matrix (Figure S2a). Thus, the peaks at 2917 cm^−1^ and 1730 cm^−1^ in the FT-IR DA-CIT-SLNs-60 spectrum could be ascribed to partially hydrated Gelucire^®^ 50/13 [42] and, in particular, the latter peak should be attributable to the ester carbonyl function of the Gelucire^®^ 50/13, whereas the absorption bands at 1656 cm^−1^ and 1472 cm^−1^ of the FT-IR spectrum of DA-CIT-SLNs-60 are not of unequivocal attribution. Overall, it may be stated that the FT-IR spectrum of DA-CIT-SLNs-60 shows absorption bands attributable to the lipid matrix, whereas those due to DA and CIT cannot be easily detected. This may probably be due to the fact that the characteristic absorption bands of the neurotransmitter DA and antioxidant CIT are of weaker intensity compared to those of Gelucire^®^ 50/13 and, hence, are covered from these last ones.

#### 3.3.2. DSC Analysis

As previously reported [22], the DSC thermogram of pure CIT (Figure S2b) showed two endothermic peaks at 130 °C and 150 °C, together with an exothermic peak at about 260 °C. This last peak should be due to melting with exothermic decomposition of CIT, because it is reported that the melting point of this active substance is in the range 259–268 °C [19]. As for the presence of two endothermic peaks at 130 °C and 150 °C in the same DSC profile, in our opinion, this may be due to the presence of two different solvates (hydrates) since CIT is a hygroscopic powder and stable hydrates may be formed [43]. In the DSC thermogram of DA-CIT-SLNs-60 (Figure S2b), a broad exothermic peak is present at 150 °C and an endothermic one at 210 °C. Concerning such DSC profile, our hypothesis is that the broad exothermic peak at 150 °C may be due to the melting with exothermic decomposition of CIT, and the endothermic one at 210 °C may be attributable to the melting of the neurotransmitter DA. Both these thermal processes, indeed, may be considerably shifted at lower temperatures when DA and CIT are co-loaded in SLNs. It is clear that our hypotheses remain to be demonstrated, and this could be the topic of future work aiming to assess such thermoanalytical outcomes. Overall, it can be stated that a marked reduction in crystallinity occurs once the active substances herein studied are co-encapsulated in the SLN structure.

#### 3.3.3. X-ray Diffraction Spectra

The XRPD diffractograms of pure DA (Figure S2c left) and CIT [22] clearly demonstrate their crystalline nature. The diffractograms of DA-SLNs and DA-CIT-SLNs-60 (Figure S2c middle and right) show similar XRPD patterns, with the most intense peaks at 2θ 19 (°) and 2θ 23 (°) due to the SLN matrix [22], and a peak at 2θ 37.6 (°) which could be ascribed only to the DA crystal structure, since at such 2θ values, only diffraction peaks of little intensity occur in the spectrum of pure CIT. Concerning DA-CIT-SLNs-60, the absence of any XRPD reflection of CIT suggests a most reduced crystallinity of the antioxidant compound once both the active substances are nanoencapsulated in DA-CIT-SLNs-60 (Figure S2c right).

### 3.4. In Vitro Release Studies of DA-CIT-SLNs-60

Reported in Figure 3 are the release profiles of the neurotransmitter and the antioxidant from DA-CIT-SLNs-60 by using SNF enriched with mucin to better mimic the liquid nasal compartment as release medium. As shown, when the release of CIT was examined in SNF from the studied nanocarriers, after a burst up to 5 h leading to 9% of the total antioxidant delivered, a progressive decrease in the antioxidant released was noticed up to 24 h when the study was stopped. On the other hand, an almost linear sustained release kinetic of the neurotransmitter was observed over time, reaching 10% of DA delivered after 24 h.

### 3.5. Cytobiocompatibility in SH-SY5Y and RPMI 2650 Cells

**Figure 4:**
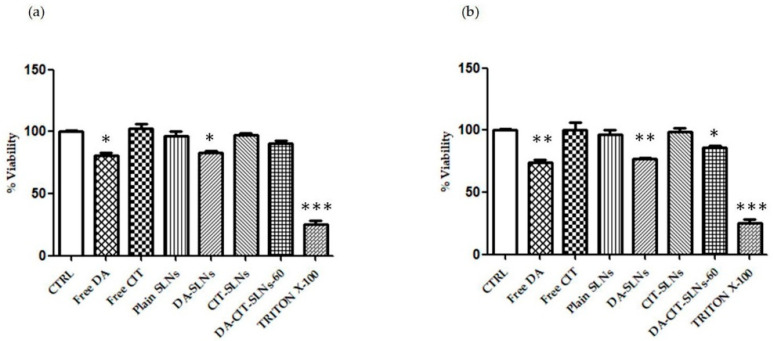
SHSY-5Y cells were challenged with free DA, free CIT, DA-SLNs, CIT-SLNs, DA-CIT-SLNs-60, and plain SLNs for 24 h, considering (**a**) 50 μM DA and 11.5 μM CIT and (**b**) 100 μM DA and 23 μM CIT. Cells were then assayed for vitality using the resazurin assay. Control (CTRL) cells are untreated cells (100% of vitality), whereas TX-100 (0.1% Triton X-100) denotes positive controls. Data are expressed as average ± SD of two experiments carried out each in six wells. * *p* < 0.05, ** *p* < 0.01, *** *p* < 0.001 for all conditions vs. untreated cells.

For cytocompatibility assessment, SH-SY5Y neuronal cells were treated with free DA, free CIT, DA-SLNs, CIT-SLNs, DA-CIT-SLNs-60, and plain SLNs and viability, by keeping the doses of 100 μM and 50 μM of DA (unencapsulated or in SLNs). Such concentration values were chosen based on previous experiments that evaluated the range in which a reduced viability was observed [44,45]. Interestingly, the cytotoxic effect of DA was counteracted in the presence of CIT in both conditions, since there was not a significant difference in viability between cells treated with DA-CIT-SLNs-60 and untreated cells (Figure 4a,b).

**Figure 5:**
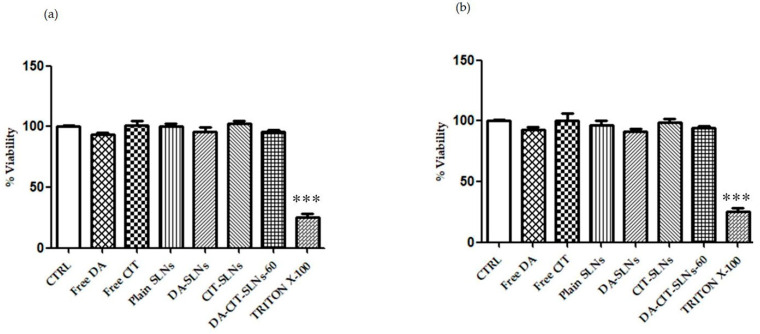
RPMI 2650 cells were challenged with free DA, free CIT, DA-SLNs, CIT-SLNs, DA-CIT-SLNs-60, and plain SLNs for 24 h, considering (**a**) 50 μM DA and 11.5 μM CIT and (**b**) 100 μM DA and 23 μM CIT. Cells were then assayed for vitality using the resazurin assay. Control (CTRL) cells are untreated cells (100% of vitality), whereas TX-100 (0.1% Triton X-100) denotes positive controls. Data are expressed as the average ± SD of two experiments carried out each in six wells. *** *p* < 0.001 for the different treatments vs. untreated cells.

Interestingly, when RPMI 2650 cells were challenged under the same conditions, no significant reduction in viability was observed at both DA doses adopted (Figure 5a,b).

### 3.6. Permeation Studies across RPMI-2650 Cell Monolayer

For the transcellular permeation study, we seeded and cultured RPMI 2650 cells on Transwell plates. Once the cells completely covered the culture insert, we then added the samples (i.e., free DA, free CIT, DA-SLNs, CIT-SLNs, and DA-CIT-SLNs-60) in the apical side. After 3, 6, and 24 h of incubation, we analyzed the concentrations of the active principles DA and CIT in the basolateral sides. The results in Table 2 demonstrate that, as expected, free DA was not able to cross the cell monolayer, whereas the DA apparent permeability coefficient (P*app*) significantly increased from DA-SLNs (P*app* = 0.0109 (±0.040 × 10^−4^) cm/s) to DA-CIT-SLNs-60 (P*app* = 0.0369 (±0.041 × 10^−4^) cm/s). In the case of the antioxidant CIT, a slight increase in permeability was revealed from the unencapsulated (P*app* = 0.0413 (±6 × 10^−8^) cm/s) to the entrapment in the CIT-SLNs (P*app* = 0.0577 (±6 × 10^−8^) cm/s). On the other hand, from DA-CIT-SLNs-60, CIT permeation took place under the HPLC limit of quantification, maybe because CIT was mainly consumed according to a redox reaction with DA, whose chemical structure was let unmodified.

### 3.7. DPPH Test and Antioxidant Activity

The prepared SLNs were evaluated for their antioxidant activity by using a slightly modified spectrophotometric method based on the use of 2,2-diphenyl-1-picrylhydrazyl (DPPH) free radical enabling to assess the ability of chemical substances to act as free radical scavengers or hydrogen donors (Table 3). As shown, a synergic effect could be deduced between pure CIT and plain SLNs because, once DA-CIT-SLNs-60 are formulated, then they result in 93.2 ± 5.0% of antioxidant activity, namely, significantly more than the control Gelucire^®^ 50/13 (** *p* ≤ 0.001). Interestingly, when the antioxidant activity of CIT alone was compared with that from CIT-SLNs and DA-CIT-SLNs-60, no statistical difference was noticed in terms of protection from oxidative stress (*p* ≥ 0.001), meaning that the nanoencapsulation process did not alter the antioxidant role exerted by CIT. Furthermore, the in vitro antioxidant activity of CIT-SLNs and DA-CIT-SLNs-60 was almost comparable from the presence of DA (93.2 ± 5.0 and 91.3 ± 1.3, for CIT-SLNs and DA-CIT-SLNs-60, respectively). However, it should be pointed out that the antioxidant activity of DA-CIT-SLNs-60 resulted in a higher statistically significant manner than CIT alone.

### 3.8. OxyBlot Assay in SHSY-5Y Cell Line

**Figure 6:**
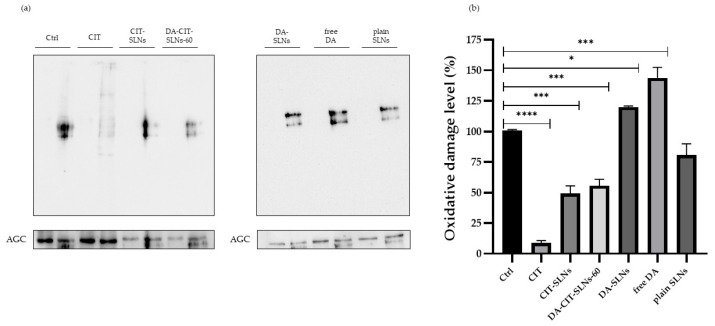
(**a**) Representative OxyBlot analysis of cell lysates obtained from untreated SHSY-5Y cells (Ctrl), and cells treated with free DA (11.50 μg/mL), CIT (7.65 μg/mL), DA-SLNs, CIT-SLNs, and DA-CIT-SLNs-60. For each lysate, the first and second lanes represent nonderivatized control and derivatized sample, respectively. Equal loading of cell lysate samples was confirmed using anti-AGC antibody. (**b**) Densitometric analysis was performed to quantify the bands, and the data were normalized to the loading control AGC. Results are expressed as mean ± SD from at least three independent experiments. * *p* ≤ 0.05, *** *p* ≤ 0.01, ****p ≤ 0.001 vs. Ctrl.

It is known that oxidative stress can damage biological macromolecules, since proteins are among the principal targets, and the quantification of protein carbonylation levels represents a key biomarker of oxidative damage. Protein carbonylation introduces carbonyl groups (aldehydes and ketones) specifically in proline, arginine, threonine, or lysine residues [46]. We determined the post-translational modification of cellular proteins through OxyBlot analysis to investigate the effect of DA (free or encapsulated, at the concentration of 11.50 μg/mL) and CIT (free or encapsulated, at the concentration of 7.65 μg/mL) on protein oxidation after 24 h of treatment. Figure 6a,b illustrate the results obtained.

In SHSY-5Y neuroblastoma cells, treatment with free DA or DA-SLNs led to high levels of carbonylated proteins, indicating the evident pro-oxidant activity of DA. Conversely, cells treated with free CIT, CIT-SLNs, and DA-CIT-SLNs-60 exhibited fewer carbonylated proteins. Furthermore, it was consistently observed that if the concentrations of DA and CIT were increased while maintaining the same concentration ratio, a protective effect of CIT was consistently observed.

## 4. Discussion

Nowadays, the development of novel multifunctional nanomedicines, which combine multiple biological roles into a single nanocarrier, has attracted the most attention for the management of neurological disorders [15]. The present work was aimed at the technological and biological evaluation of the DA-CIT-SLNs nanosystems for intranasal administration as a potential innovative treatment of PD. In these multifunctional nanomedicines, the DA should replace the loss of the neurotransmitter occurring in Parkinsonian patients, CIT should combat the oxidative stress and mitochondrial dysfunction that play a key role in PD pathogenesis, and the nasal administration should offer a noninvasive bypass of the BBB [10,11,20].

**Figure 7:**
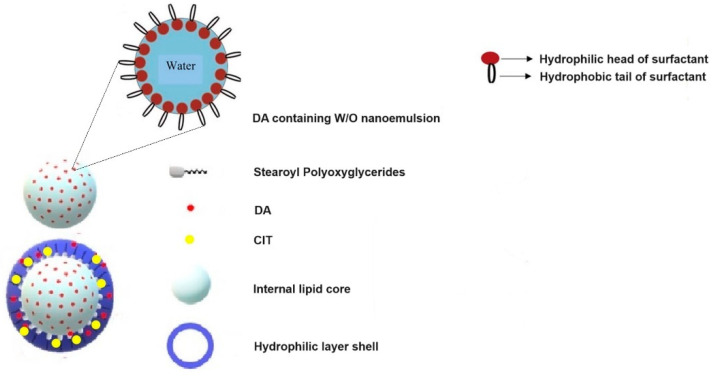
Schematic representation of the Gelucire^®^ 50/13-based DA-CIT-SLNs.

To achieve SLNs co-administering DA and CIT, we used the preparative method of SLNs developed by us, enabling the encapsulation of both lipophilic and hydrophilic active principles employing the Gelucire^®^ 50/13 as lipid matrix. With pleasure, we observed that, overall, the method also worked well in the challenging case herein examined, because both the hydrophilic actives were encapsulated each, with satisfactory encapsulation efficiency. We started varying the initial lipid amount in order to check whether it could exert some effects in terms of particle size and encapsulation efficiency. Since bigger SLNs with lower contents in DA and CIT were prepared at higher Gelucire^®^ 50/13 amounts (Table 1), we chose to perform our investigation employing Gelucire^®^ 50/13 at lower amounts (i.e., DA-CIT-SLNs-60). By comparing the results herein obtained with those previously reported in the nanoencapsulation of DA and GSE in Gelucire^®^ 50/13-based SLNs [21], we noted a marked increase in E.E.% of neurotransmitter and antioxidant in the combination of the present study (77 ± 7% and 75 ± 2%, respectively, for DA and CIT vs. 62 ± 4% and 10 ± 0%, respectively, for DA and GSE combination). Another crucial ratio that needs to be optimized for appropriate treatment of PD is the DA/CIT encapsulation molar ratio, which, in this study, was selected by us at 3/1 (mol/mol), as a starting point. It is clear that, in this regard, an appropriate experimental design should be adopted to know how easily this ratio can be tuned. However, this investigation should be the topic of future studies. A further issue that should be addressed concerns the possible location of the two hydrophilic active principles inside the SLNs. Taking into account the mentioned composition of Gelucire^®^ 50/13 (i.e., a lipid mixture able to self-emulsify in contact with aqueous media) as well as some hints from the literature [47,48,49], we proposed a model for such PEGylated SLNs [25] which we can employ to gain insights into the possible location of the two hydrophilic active principles within such PEGylated SLNs. In particular, these nanocarriers should be constituted by a hydrophilic shell comprising the polyoxyethylene chains and cosurfactant (Tween 85), together with an internal lipid layer formed by the stearoyl moieties (Figure 7). In such an SLN model, hydrophilic substances such as DA and CIT could be adsorbed on the particle surface or entrapped according to the so-called drug-enriched shell model. However, a hydrophilic substance such as DA (calculated log Po/w = −1) could be also entrapped in the aqueous phase of the W/O nanoemulsion present in the lipid core due to the ability of Gelucire^®^ 50/13 to self-emulsify in contact with aqueous media, and this corresponds to the drug-enriched core model. On the other hand, for a strongly hydrophilic substance such as CIT (calculated log Po/w = −4) and characterized by a higher number of hydrogen bonding acceptor (HA)/donor (HB) groups than DA (11 HA + 4 HB vs. 3 HA + 3 HB for DA and CIT, respectively, as computed by Cactvs 3.4.8.18 (PubChem release 2021.10.14)), an almost complete location in the hydrophilic shell should occur only because, in this layer, efficient hydrogen bonding and polar interactions could be established between the polyoxyethylene chains of Gelucire^®^ 50/13 and CIT.

Such substance localization could also explain the finding of size increase and heterogeneously distributed SLNs at higher Gelucire concentrations, as occurs for DA-CIT- SLNs-120. Indeed, at higher Gelucire concentrations, it is possible that the stability of the nanoemulsion decreased due to destabilization phenomena like creaming, sedimentation, and coalescence, leading to bigger droplet size. In support of the proposed locations, we invoke also the outcomes of the in vitro release studies on DA-CIT-SLNs (Figure 3). Indeed, the observed burst effect in the release kinetic of CIT from such nanocarriers is consistent with a location of this antioxidant agent in the hydrophilic shell of the nanoparticles. The observed decrease in CIT release after 5 h may be due to the CIT degradation products ascribable to hydrolytic and/or oxidative processes [50]. Similarly, the almost linear sustained release of DA found for DA-CIT-SLNs is also consistent with a neurotransmitter location as nanoemulsion within the lipid core of the examined PEGylated SLNs. On the other hand, this different release behavior suggests that the co-encapsulation is not always more advantageous than the formulation of two different SLNs. Indeed, at longer times, when more neurotransmitter is released, the amount of the antioxidant agent is lower, with the risk of toxicity issues that may arise. This is a possible drawback of using such multifunctional nanoformulations. Overall, such findings corroborate the assessment that the preparative method of SLNs based on the self-emulsifying lipid Gelucire^®^ 50/13 constitutes a satisfactory procedure for entrapping not only one, but also two hydrophilic active principles, such as DA and CIT. Moreover, such a preparative method is essentially organic solvent free, in addition to the negligible amount of AcOH solution (0.01%, w/v) added to avoid pH values of the medium higher than 6, where the spontaneous DA autoxidation is markedly favored [51]. Lastly, this method of producing SLNs also appears suitable for obtaining a satisfactory development towards industrial scale-up employing microfluidic tools [52]. A DA controlled release system should meet three main conditions to maintain this option viable: (1) allow stabilization in its basal state; (2) facilitate its release under physiological conditions; (3) this release should take place in the necessary regions, particularly in the substantia nigra pars compacta [53]. Since DA is a highly reactive molecule, a novel concept regarding its administration exploits the use of co-administration of antioxidants, such as in is our case of CIT. In general, increased stability of ingredients has been observed when co-encapsulated with antioxidant reagents, reaching higher functionality and bioactivity compared to a single bioactive by inducing synergistic effects between ingredients [54]. The release kinetics and permeation studies presented herein may suggest that upon in vivo administration, a more prolonged release of CIT would be necessary to avoid DA autoxidation, which is well known to be responsible for neurotoxicity. Thus, novel formulations should be exploited to increase CIT content. CIT was not toxic at the doses used in our experimental model in vitro, but it would be possible to increase the doses in in vivo experiments without observing any toxic effects, since it has been documented that, when administered orally or by injection, CIT is nontoxic and is very well tolerated [55]. Furthermore, different studies were performed in vivo in order to assess the acute, subacute, and chronic toxicity of citicoline [56]. On the other hand, DA’s controlled release would allow its use according to the needs of the dopaminergic neurons without causing an overdose that could lead to dysregulation of motor performance [53].

As for the physical stability of DA-CIT-SLNs, based on their zeta potential values, such nanocarriers were expected to be of moderate stability. Indeed, when monitoring the particle size at 4 °C for a week and at 37 °C for 24 h, moderate stability was practically observed for the studied nanosystems (Figure 2). However, an intriguing behavior noted was that, when comparing the outcomes at 4 °C for one day with those at 37 °C for 24 h, it resulted that, in the latter conditions, the particle size increase was lower than that observed in the former ones, leading to the conclusion that the studied nanosystems should be more physically stable at higher temperatures. To account for this unusual behavior, our opinion is that, in both conditions examined, the nanocarriers tend to aggregate in agreement with the low negative surface charge measured. However, such an aggregation process is slower at 37 °C compared to 4 °C, due to the increased thermal movements, which hinder this nanoparticle accumulation phenomenon.

Concerning the solid-state studies on DA-CIT-SLNs, the relative results, taken together, indicate that a reduction in the crystalline solid state of CIT and DA occurs when they are co-encapsulated in DA-CIT-SLNs-60 (Figure S2). This outcome parallels with the DA-SLNs solid state (Figure S2b) and with the CIT-SLNs solid state [22].

From a biological viewpoint, cytocompatibility was observed for DA-CIT-SLNs-60, according to the resazurin assay when both RPMI 2650 and SH-SY5Y cells were incubated with SLN formulations, which did not cause any cell toxicity (Figure 4a,b and Figure 5a,b). In the presence of both cell lines, we worked with 50 and 100 μM at most of free or encapsulated DA, following the suggestion from the literature indicating that DA concentrations higher than 100 μM for SH-SY5Y were found to be toxic enough within 24 h [57]. However, the resazurin-based colorimetric assay could be biased by the color change accompanying neurotransmitter decomposition. A more accurate evaluation of nanoparticle cytotoxicity can be achieved using real-time cell analysis (RTCA), which is an impedance-based assay that minimizes the interference issue with optically active nanoparticles, enables the evaluation of higher concentrations, and manifests the real-time capability of screening nanoparticle cytotoxicity [58,59,60]. On the other hand, focusing on P*app* values and permeation studies from DA-CIT SLNs-60, within 24 h, DA, unlike free form, was found intact in the basolateral compartment at each time point. It may be interpreted that the CIT present in DA-CIT SLNs-60 was also capable to protect DA from early degradation during permeation across the RMPI 2650 cell monolayer (Table 2), while this did not occur for the permeation of the free form. Moreover, an enhancement of DA P*app* resulted from DA-CIT SLNs-60 in comparison to DA-SLNs (0.0369 (±0.041 × 10^−4^) cm/s vs. 0.0109 (±0.040 × 10^−4^) cm/s), respectively, Table 2). Such a P*app* increase can be explained by taking into account that the role of SLNs as a penetration enhancer of nasal mucosa has been already shown in the literature [61]. In addition to this, we hypothesized that the DA-CIT-SLNs-60 permeation across cell membranes of the human nasal epithelium of RPMI 2650 cells is enhanced also because they possess a smaller particle size than DA-SLNs (Table 1). Furthermore, gaining insight into the permeation across the RPMI 2650 barrier, from Table 2, it is apparent that free CIT and CIT from CIT-SLNs were seen to permeate across the human nasal barrier of the RMPI 2650 cell monolayer in both cases. Interestingly, however, from DA-CIT SLNs-60, the antioxidant compound in the basolateral compartment was below the limit of quantification (LOQ) of the HPLC analytical method employed (see Section 2.3.). This very low amount of CIT permeated, in our opinion, may be mostly a consequence of the redox reaction that takes place between CIT and DA, leading to consumption of the antioxidant agent. In addition to this, it should be also considered that, to some extent, further CIT loss may also occur during sample manipulations because of an almost complete location of the antioxidant in the hydrophilic shell during sample manipulations. Lastly, the observed significantly higher antioxidant activity of DA-CIT-SLNs-60 than CIT alone (Table 3), can be also related to the abovementioned role of SLNs as a penetration enhancer of nasal mucosa.

To better quantify the oxidative cellular damage level in the SHSY-5Y neuronal cells of free and nanoencapsulated substances herein studied, the OxyBlot test was performed (Figure 6). The imbalance between pro-oxidant and antioxidant systems is a condition observed in neurodegenerative diseases. Several studies have pointed out that this state of oxidative stress significantly contributes to the progression of the mentioned diseases [62]. One particular aspect is the oxidative modification of proteins, which has been found to play a pivotal role in several pathological processes. Oxidation mainly alters the chemical properties of amino acid side chains, leading to structural and folding changes in proteins. In many instances, these modifications result in protein impairment, although some modifications may instead activate the proteins [63]. A previous work revealed that in the neurodegeneration of PD, reactive oxygen species (ROS) induced carbonyl modification of a molecular chaperone, heat shock 70-kDa protein 1 (Hsp70.1), especially in its key site, Arg469 [64]. Finally, in our recent study, we demonstrated that in human neuroblastoma SH-SY5Y cells, a high accumulation of carbonylated proteins is observed in the presence of free DA [65].

The OxyBlot assay (Figure 6) shows a significant increase in cellular oxidative damage (expressed in terms of protein carbonylation) in SHSY-5Y neuroblastoma cells when treated with DA-SLNs compared to the same cells treated with free CIT or DA-CIT-SLNs-60, which demonstrates a significant protective effect due to the antioxidant CIT.

To perform a more in-depth analysis of such results, by comparing the data from the DPPH test, aimed at evaluating the in vitro antioxidant effect (Table 3), with OxyBlot output, assessing the protective effect from oxidative damage, the following rank orders were obtained. From DPPH test: CIT-SLNs = DA-CIT-SLNs-60 > free CIT, whereas from OxyBlot assay (Figure 6b), the rank order for the protective effect was free CIT > CIT-SLNs = DA-CIT-SLNs-60. The observed discrepancy between antioxidant alone and its formulations could be ascribable to the fact that OxyBlot measurements, unlike DPPH ones, are performed in the presence of a delicate cell model line, namely, the neuronal SHSY-5Y cells. However, the DA-CIT-SLN-60 formulation only possesses the advantage of a multifunctional nanomedicine, i.e., to combat the oxidative stress and mitochondrial dysfunction providing neurotransmitter to replace its loss in Parkinsonian patients.

## 5. Conclusions

From a technological point of view, an important conclusion of the present study is that the preparative method of SLNs based on the self-emulsifying lipid Gelucire^®^ 50/13 as lipid matrix works well in terms of encapsulation efficiencies even when two hydrophilic substances such as DA and CIT are to be encapsulated. Such a preparative method is essentially organic solvent free and may constitute a valid alternative to the usual double emulsification (W1/O/W2) strategy employed in such circumstances. As for biological aspects, in view of an intranasal administration of DA-CIT-SLNs-60, the co-administration of DA and CIT could be beneficial under different points of view. Firstly, the release of CIT from the studied SLNs can be addressed to restore neuron functionality into the brain of PD patients. Secondly, another benefit relies on the fact that, thanks to the presence of CIT, the oxidative stress and mitochondrial dysfunction are lowered, and this contributes to limiting the progression of PD. In this regard, it is important to underline the considerable potential of the OxyBlot assay to gain insights on the protective effect of antioxidant agents towards oxidative stress and mitochondrial dysfunction. Notably, the platform herein evaluated, i.e., DA-CIT-SLNs-60, can be appropriately adapted for application to other neurological diseases.

## Figures and Tables

**Table 1:** Physicochemical properties of SLNs studied ^a^.

Formulation	Size (nm)	PDI ^b^	Zeta Potential (mV)	E.E. DA (%)	E.E. CIT (%)
DA-CIT-SLNs-60	131 ± 20	0.40 ± 0.04	−10.2 ± 1.1	77 ± 7	75 ± 2
DA-CIT-SLNs-120	405 ± 25 **	0.53 ± 0.01	−7.8 ± 0.4	65 ± 3	59 ± 8
DA-SLNs ^c^	171 ± 6 **	0.20 ± 0.01	−2.0 ± 0.7 **	19 ± 3	-
CIT-SLNs ^d^	201 ± 24 **	0.45 ± 0.08	−2.2 ± 0.2 **	-	80 ± 7
Plain SLNs ^e^	141 ± 11	0.35 ± 0.17	−9.7 ± 0.8	-	-

^a^ Mean ± standard deviation of at least eight replicates (n = 8) are reported. Plain SLNs were taken as control for statistical evaluation. ^b^ PDI: polydispersity index. ^c^ From Reference [25]; ^d^ From Reference [22]; ^e^ From Reference [39]. ** *p* ≤ 0.001 as compared with plain SLNs.

**Table 2:** Apparent permeability coefficient (P*app*) of DA and CIT, each one unencapsulated and from SLNs, across RPMI 2650 cells.

Formulation	P*app* DA (cm/s)	P*app* CIT (cm/s)
DA-CIT-SLNs-60	0.0369 (±0.041 × 10^−4^)	<LOQ
DA-SLNs	0.0109 (±0.040 × 10^−4^)	-
CIT-SLNs	-	0.0577 (±6 × 10^−8^)
Free-DA	NP ^a^	
Free CIT		0.0413 (±6 × 10^−8^)

^a^ NP: No permeation. Data are mean ± standard deviation of six replicates (n = 6).

**Table 3:** In vitro antioxidant activity evaluated according to DPPH test.

Formulation	Antioxidant Activity (%)
CIT	88.6 ± 0.6
Gelucire^®^50/13 ^a^	55.0 ± 0.2
Plain SLNs ^a^	72.8 ± 5.3
CIT-SLNs	93.2 ± 5.0
DA-SLN ^a^	54.7 ± 2.5
DA-CIT-SLNs-60	91.3 ± 1.3

^a^ From Ref. [30]. Data are mean ± standard deviation of six replicates.

## Data Availability

Data are contained within the article and Supplementary Materials.

## Supplementary Materials

The following supporting information can be downloaded at: https://www.mdpi.com/article/10.3390/pharmaceutics16081048/s1, Figure S1: Particle size distribution of DA-CIT-SLNs-60 (a) and DA-CIT-SLNs-120 (b); Figure S2: Panel (a): FT-IR spectra of pure DA (a), pure CIT (b), DA-SLNs (c), and DA-CIT-SLNs-60 (d). Panel (b): DSC profiles of pure DA (a), pure CIT (b), DA-SLNs (c), CIT-SLNs (d), and DA-CIT-SLNs-60 (e). Panel (c): X-ray diffraction patterns of free DA (left), DA-SLNs (middle), and DA-CIT-SLNs-60 (right). For the FT-IR spectra of CIT-SLNs and the X-ray diffraction patterns of free CIT and CIT-SLNs, the reader is referred to Ref. [22].

## References

[1] Al-Kuraishy H.M., Al-Gareeb A.I. (2020). Citicoline Improves Human Vigilance and Visual Working Memory: The Role of Neuronal Activation and Oxidative Stress. Basic Clin. Neurosci..

[2] Abdel-Salam O.M.E., Youness E.R., Mohammed N.A., Abd El-Moneim O.M., Shaffie N. (2019). Citicoline Protects against Tramadol-Induced Oxidative Stress and Organ Damage. React. Oxyg. Species.

[3] Mozafari N., Farjadian F., Mohammadi Samani S., Azadi S., Azadi A. (2020). Simvastatin-chitosan-citicoline conjugates nanoparticles as the co-delivery system in Alzheimer susceptible patients. Int. J. Biol. Macromol..

[4] Que D.S., Jamora R.D.G. (2021). Citicoline as Adjuvant Therapy in Parkinson’s Disease: A Systematic Review. Clin. Ther..

[5] Agulla J., Brea D., Campos F., Sobrino T., Argibay B., Al-Soufi W., Blanco M., Castillo J., Ramos-Cabrer P. (2013). In vivo theranostics at the peri-infarct region in cerebral ischemia. Theranostics.

[6] Wold Health Organization (WHO). Over 1 in 3 People Affected by Neurological Conditions, the Leading Cause of Illness and Disability Worldwid. http://www.who.int..

[7] Kaur G., Arora M., Ravi Kumar M.N.V. (2019). Oral Drug Delivery Technologies-A Decade of Developments. J. Pharmacol. Exp. Ther..

[8] Trapani G., Franco M., Trapani A., Lopedota A., Latrofa A., Gallucci E., Micelli S., Liso G. (2004). Frog intestinal sac: A new in vitro method for the assessment of intestinal permeability. J. Pharm. Sci..

[9] Keller L.A., Merkel O., Popp A. (2022). Intranasal drug delivery: Opportunities and toxicologic challenges during drug development. Drug Deliv. Transl. Res..

[10] Crowe T.P., Hsu W.H. (2022). Evaluation of Recent Intranasal Drug Delivery Systems to the Central Nervous System. Pharmaceutics.

[11] Koo J., Lim C., Oh K.T. (2024). Recent Advances in Intranasal Administration for Brain-Targeting Delivery: A Comprehensive Review of Lipid-Based Nanoparticles and Stimuli-Responsive Gel Formulations. Int. J. Nanomed..

[12] Samal J., Rebelo A.L., Pandit A. (2019). A window into the brain: Tools to assess pre-clinical efficacy of biomaterials-based therapies on central nervous system disorders. Adv. Drug Deliv. Rev..

[13] Singh A., Kutscher H.L., Bulmahn J.C., Mahajan S.D., He G.S., Prasad P.N. (2020). Laser ablation for pharmaceutical nanoformulations: Multi-drug nanoencapsulation and theranostics for HIV. Nanomedicine.

[14] Ancona A., Sportelli M., Trapani A., Picca R.A., Palazzo C., Bonerba E., Mezzapesa F., Tantillo G., Trapani G., Cioffi N. (2014). Synthesis and Characterization of Hybrid Copper-Chitosan Nano-antimicrobials by Femtosecond Laser-Ablation in Liquids. Mater. Lett..

[15] Faria P., Pacheco C., Moura R.P., Sarmento B., Martins C. (2023). Multifunctional nanomedicine strategies to manage brain diseases. Drug Deliv. Transl. Res..

[16] Park M.W., Cha H.W., Kim J., Kim J.H., Yang H., Yoon S., Boonpraman N., Yi S.S., Yoo I.D., Moon J.S. (2021). NOX4 promotes ferroptosis of astrocytes by oxidative stress-induced lipid peroxidation via the impairment of mitochondrial metabolism in Alzheimer’s diseases. Redox Biol..

[17] Rodriguez-Nogales C., Garbayo E., Carmona-Abellan M.M., Luquin M.R., Blanco-Prieto M.J. (2016). Brain aging and Parkinson’s disease: New therapeutic approaches using drug delivery systems. Maturitas.

[18] Bonferoni M.C., Rassu G., Gavini E., Sorrenti M., Catenacci L., Giunchedi P. (2020). Nose-to-Brain Delivery of Antioxidants as a Potential Tool for the Therapy of Neurological Diseases. Pharmaceutics.

[19] Qadri R., Goyal V., Behari M., Subramanian A., Datta S.K., Mukhopadhyay A.K. (2021). Alteration of Mitochondrial Function in Oxidative Stress in Parkinsonian Neurodegeneration: A Cross-Sectional Study. Ann. Indian Acad. Neurol..

[20] Wen P., Ren C. (2024). Research progress on intranasal treatment for Parkinson’s disease. Neuroprotection.

[21] Trapani A., Castellani S., Guerra L., De Giglio E., Fracchiolla G., Corbo F., Cioffi N., Passantino G., Poeta M.L., Montemurro P. (2023). Combined Dopamine and Grape Seed Extract-Loaded Solid Lipid Nanoparticles: Nasal Mucosa Permeation, and Uptake by Olfactory Ensheathing Cells and Neuronal SH-SY5Y Cells. Pharmaceutics.

[22] Margari A., Monteduro A.G., Rizzato S., Capobianco L., Crestini A., Rivabene R., Piscopo P., D‘Onofrio M., Manzini V., Trapani G. (2022). The Encapsulation of Citicoline within Solid Lipid Nanoparticles Enhances Its Capability to Counteract the 6-Hydroxydopamine-Induced Cytotoxicity in Human Neuroblastoma SH-SY5Y Cells. Pharmaceutics.

[23] Satapathy M.K., Yen T.L., Jan J.S., Tang R.D., Wang J.Y., Taliyan R., Yang C.H. (2021). Solid Lipid Nanoparticles (SLNs): An Advanced Drug Delivery System Targeting Brain through BBB. Pharmaceutics.

[24] Trapani A., Mandracchia D., Tripodo G., Di Gioia S., Castellani S., Cioffi N., Ditaranto N., Esteban M.A., Conese M. (2019). Solid lipid nanoparticles made of self-emulsifying lipids for efficient encapsulation of hydrophilic substances. AIP Conference Proceedings.

[25] Trapani A., De Giglio E., Cometa S., Bonifacio M.A., Dazzi L., Di Gioia S., Hossain M.N., Pellitteri R., Antimisiaris S.G., Conese M. (2021). Dopamine-loaded lipid based nanocarriers for intranasal administration of the neurotransmitter: A comparative study. Eur. J. Pharm. Biopharm..

[26] De Giglio E., Bakowsky U., Engelhardt K., Caponio A., La Pietra M., Cometa S., Castellani S., Guerra L., Fracchiolla G., Poeta M.L. (2023). Solid Lipid Nanoparticles Containing Dopamine and Grape Seed Extract: Freeze-Drying with Cryoprotection as a Formulation Strategy to Achieve Nasal Powders. Molecules.

[27] Cometa S., Bonifacio M.A., Trapani G., Di Gioia S., Dazzi L., De Giglio E., Trapani A. (2020). In vitro investigations on dopamine loaded Solid Lipid Nanoparticles. J. Pharm. Biomed. Anal..

[28] Trapani A., De Giglio E., Cafagna D., Denora N., Agrimi G., Cassano T., Gaetani S., Cuomo V., Trapani G. (2011). Characterization and evaluation of chitosan nanoparticles for dopamine brain delivery. Int. J. Pharm..

[29] Trapani A., Cometa S., De Giglio E., Corbo F., Cassano R., Di Gioia M.L., Trombino S., Hossain M.N., Di Gioia S., Trapani G. (2022). Novel Nanoparticles Based on N,O-Carboxymethyl Chitosan-Dopamine Amide Conjugate for Nose-to-Brain Delivery. Pharmaceutics.

[30] Trapani A., Corbo F., Agrimi G., Ditaranto N., Cioffi N., Perna F., Quivelli A., Stefano E., Lunetti P., Muscella A. (2021). Oxidized Alginate Dopamine Conjugate: In Vitro Characterization for Nose-to-Brain Delivery Application. Materials.

[31] Stephenson A.P., Schneider J.A., Nelson B.C., Atha D.H., Jain A., Soliman K.F., Aschner M., Mazzio E., Renee Reams R. (2013). Manganese-induced oxidative DNA damage in neuronal SH-SY5Y cells: Attenuation of thymine base lesions by glutathione and N-acetylcysteine. Toxicol. Lett..

[32] Wan X., Wang W., Liang Z. (2021). Epigallocatechin-3-gallate inhibits the growth of three-dimensional in vitro models of neuroblastoma cell SH-SY5Y. Mol. Cell. Biochem..

[33] Yamashita K., Kiyonari S., Tsubota S., Kishida S., Sakai R., Kadomatsu K. (2020). Thymidylate synthase inhibitor raltitrexed can induce high levels of DNA damage in MYCN-amplified neuroblastoma cells. Cancer Sci..

[34] Mercier C., Hodin S., He Z., Perek N., Delavenne X. (2018). Pharmacological Characterization of the RPMI 2650 Model as a Relevant Tool for Assessing the Permeability of Intranasal Drugs. Mol. Pharm..

[35] Kreft M.E., Jerman U.D., Lasic E., Lanisnik Rizner T., Hevir-Kene N., Peternel L., Kristan K. (2015). The characterization of the human nasal epithelial cell line RPMI 2650 under different culture conditions and their optimization for an appropriate in vitro nasal model. Pharm. Res..

[36] Fir M., Milivojevic L., Prosek M., Smidovnik A. (2009). Properties Studies of Coenzyme Q10-Cyclodextrins complexes. Acta Chim. Slov..

[37] Aresta A., Calvano C.D., Trapani A., Cellamare S., Zambonin C.G., De Giglio E. (2013). Development and analytical characterization of vitamin(s)-loaded chitosan nanoparticles for potential food packaging applications. J. Nanopart. Res..

[38] Lunetti P., Di Giacomo M., Vergara D., De Domenico S., Maffia M., Zara V., Capobianco L., Ferramosca A. (2019). Metabolic reprogramming in breast cancer results in distinct mitochondrial bioenergetics between luminal and basal subtypes. FEBS J..

[39] Trapani A., Tripodo G., Mandracchia D., Cioffi N., Ditaranto N., De Leo V., Cordero H., Estebane M.A. (2018). Glutathione-loaded solid lipid nanoparticles based on Gelucire® 50/13: Spectroscopic characterization and interactions with fish cells. J. Drug Deliv. Sci. Technol..

[40] Di Gioia S., Trapani A., Cassano R., Di Gioia M.L., Trombino S., Cellamare S., Bolognino I., Hossain M.N., Sanna E., Trapani G. (2021). Nose-to-brain delivery: A comparative study between carboxymethyl chitosan based conjugates of dopamine. Int. J. Pharm..

[41] Trapani A., Mandracchia D., Tripodo G., Cometa S., Cellamare S., De Giglio E., Klepetsanis P., Antimisiaris S.G. (2018). Protection of dopamine towards autoxidation reaction by encapsulation into non-coated- or chitosan- or thiolated chitosan-coated-liposomes. Colloids Surf. B Biointerfaces.

[42] Trapani A., Guerra L., Corbo F., Castellani S., Sanna E., Capobianco L., Monteduro A.G., Manno D.E., Mandracchia D., Di Gioia S. (2021). Cyto/Biocompatibility of Dopamine Combined with the Antioxidant Grape Seed-Derived Polyphenol Compounds in Solid Lipid Nanoparticles. Molecules.

[43] Lerch H.G. (2000). Hyperhydrated Citicoline, Process and Use. U.S. Patent.

[44] Banerjee K., Munshi S., Sen O., Pramanik V., Roy Mukherjee T., Chakrabarti S. (2014). Dopamine Cytotoxicity Involves Both Oxidative and Nonoxidative Pathways in SH-SY5Y Cells: Potential Role of Alpha-Synuclein Overexpression and Proteasomal Inhibition in the Etiopathogenesis of Parkinson’s Disease. Park. Dis..

[45] Shavali S., Sens D.A. (2008). Synergistic neurotoxic effects of arsenic and dopamine in human dopaminergic neuroblastoma SH-SY5Y cells. Toxicol. Sci..

[46] Stadtman E.R. (1993). Oxidation of free amino acids and amino acid residues in proteins by radiolysis and by metal-catalyzed reactions. Annu. Rev. Biochem..

[47] Yuan H., Chen C.Y., Chai G.H., Du Y.Z., Hu F.Q. (2013). Improved transport and absorption through gastrointestinal tract by PEGylated solid lipid nanoparticles. Mol. Pharm..

[48] Matougui N., Boge L., Groo A.C., Umerska A., Ringstad L., Bysell H., Saulnier P. (2016). Lipid-based nanoformulations for peptide delivery. Int. J. Pharm..

[49] Garg G., Garg S., Patel P., Gupta G.D., Kurmi B.D. (2024). Advances in solid-lipid nanoparticle chemistry as drug delivery vehicles. Int. J. Polym. Mater. Polym. Biomater..

[50] Abdelrahman M.M., Ahmed A.B., Omar M.A., Derayea S.M., Abdelwahab N.S. (2020). Development and validation of stability indicating chromatographic methods for simultaneous determination of citicoline and piracetam. J. Sep. Sci..

[51] Umek N., Gersak B., Vintar N., Sostaric M., Mavri J. (2018). Dopamine Autoxidation Is Controlled by Acidic pH. Front. Mol. Neurosci..

[52] Monteduro A.G., Rizzato S., Caragnano G., Trapani A., Giannelli G., Maruccio G. (2023). Organs-on-chips technologies—A guide from disease models to opportunities for drug development. Biosens. Bioelectron..

[53] Padilla-Godinez F.J., Ruiz-Ortega L.I., Guerra-Crespo M. (2022). Nanomedicine in the Face of Parkinson’s Disease: From Drug Delivery Systems to Nanozymes. Cells.

[54] Shakeri M., Ghobadi R., Sohrabvandi S., Khanniri E., Mollakhalili-Meybodi N. (2024). Co-encapsulation of omega-3 and vitamin D(3) in beeswax solid lipid nanoparticles to evaluate physicochemical and in vitro release properties. Front. Nutr..

[55] Grieb P. (2014). Neuroprotective properties of citicoline: Facts, doubts and unresolved issues. CNS Drugs.

[56] Secades J.J., Gareri P. (2022). Citicoline: Pharmacological and clinical review, 2022 update. Rev. Neurol..

[57] Ganguly U., Ganguly A., Sen O., Ganguly G., Cappai R., Sahoo A., Chakrabarti S. (2019). Dopamine Cytotoxicity on SH-SY5Y Cells: Involvement of alpha-Synuclein and Relevance in the Neurodegeneration of Sporadic Parkinson’s Disease. Neurotox. Res..

[58] Saladino G.M., Kilic N.I., Brodin B., Hamawandi B., Yazgan I., Hertz H.M., Toprak M.S. (2021). Carbon Quantum Dots Conjugated Rhodium Nanoparticles as Hybrid Multimodal Contrast Agents. Nanomaterials.

[59] Tincu R., Mihaila M., Bostan M., Teodorescu F., Istrati D., Badea N., Lacatusu I. (2023). Novel Bovine Serum Albumin-Decorated-Nanostructured Lipid Carriers Able to Modulate Apoptosis and Cell-Cycle Response in Ovarian, Breast, and Colon Tumoral Cells. Pharmaceutics.

[60] Lacatusu I., Badea N., Badea G., Mihaila M., Ott C., Stan R., Meghea A. (2019). Advanced bioactive lipid nanocarriers loaded with natural and synthetic anti-inflammatory actives. Chem. Eng. Sci..

[61] Costa C.P., Barreiro S., Moreira J.N., Silva R., Almeida H., Sousa Lobo J.M., Silva A.C. (2021). In Vitro Studies on Nasal Formulations of Nanostructured Lipid Carriers (NLC) and Solid Lipid Nanoparticles (SLN). Pharmaceuticals.

[62] Qian K., Gu Y., Zhao Y., Li Z., Sun M. (2014). Citicoline protects brain against closed head injury in rats through suppressing oxidative stress and calpain over-activation. Neurochem. Res..

[63] Olufunmilayo E.O., Gerke-Duncan M.B., Holsinger R.M.D. (2023). Oxidative Stress and Antioxidants in Neurodegenerative Disorders. Antioxidants.

[64] Oikawa S., Kobayashi H., Kitamura Y., Zhu H., Obata K., Minabe Y., Dazortsava M., Ohashi K., Tada-Oikawa S., Takahashi H. (2014). Proteomic analysis of carbonylated proteins in the monkey substantia nigra after ischemia-reperfusion. Free Radic. Res..

[65] Bellucci S., Fracchiolla G., Pannunzio A., Caponio A., Donghia D., Corbo F., Capobianco L., Muscella A., Manno D.E., Stefàno E. (2023). Dopamine and Antioxidant Grape Seed Extract loaded chitosan nanoparticles: A preliminary in vitro characterization. Nano Med. Mater..

