# Supplementary material for: Dopamine and Citicoline-Co-Loaded Solid Lipid Nanoparticles as Multifunctional Nanomedicines for Parkinson’s Disease Treatment by Intranasal Administration
Source: Pharmaceutics. 2024 Aug 7;16(8):1048. doi: 10.3390/pharmaceutics16081048 (PMC11360708; doi:10.3390/pharmaceutics16081048)
Supplement: Supplementary file 1 [file pharmaceutics-16-01048-s001.zip › pharmaceutics-3123028-supplementary.pdf]

pharmaceutics

SUPPLEMENTARY INFORMATION OF

Dopamine and Citicoline-co-Loaded Solid Lipid Nanoparticles as Multifunctional Nanomedicines for Parkinson Disease Treatment by Intranasal Administration

Stefano Castellani[1,†], Giorgia Natalia Iaconisi[2,†], Francesca Tripaldi[3], Vito Porcelli[4], Adriana Trapani[3*], Eugenia Messina[4], Lorenzo Guerra[4], Cinzia Di Franco[5], Giuseppe Maruccio[6,7], Annagrazia Monteduro[6,7], Filomena Corbo[3], Sante Di Gioia[8], Giuseppe Trapani[3], and Massimo Conese[8]

[1] Department of Precision and Regenerative Medicine and Ionian Area (DiMePRe-J), University of Bari "Aldo Moro", 70125-Bari, Italy; stefano.castellani@uniba.it (S.C.)
[2] Department of Biological and Environmental Sciences and Technologies, University of Salento, 73100 Lecce, Italy; giorgianatalia.iaconisi@uniba.it (G.N.I.)
[3] Department of Pharmacy-Drug Sciences, University of Bari "Aldo Moro", 70125-Bari, f.tripaldi4@uniba.it (F.T.), giuseppe.trapani080@gmail.com (G.T.); filomena.corbo@uniba.it (F.C.); adriana.trapani@uniba.it (A.T.)
[4] Department of Biosciences, Biotechnologies and Environment, University of Bari "Aldo Moro", 70125-Bari, Italy; lorenzo.guerra1@uniba.it (L.G.); vito.porcelli@uniba.it (V.P.); eugenia.messina@uniba.it (E.M.)
[5] CNR-IFN Bari, Bari Via Amendola 173, 70126 Bari, Italy;cinzia.difranco@cnr.it (C.D.)
[6] Omnics Research Group, Department of Mathematics and Physics "Ennio De Giorgi", University of Salento and INFN Sezione di Lecce, Via per Monteroni, 73100 Lecce, Italy; annagrazia.monteduro@unisalento.it (A.G.M.)
[7] CNR-NANOTEC Institute of Nanotechnology, Via per Monteroni, 73100 Lecce, Italy; giuseppe.maruccio@unisalento.it (G.M.); annagrazia.monteduro@unisalento.it (A.G.M.).
[8] Department of Clinical and Experimental Medicine, University of Foggia, 71122 Foggia, Italy; sante.digioia@unifg.it (S.D.G); massimo.conese@unifg.it (M.C.)
* Correspondence: adriana.trapani@uniba.it; Tel.: +39-080-544211
† These authors contributed equally to this work.

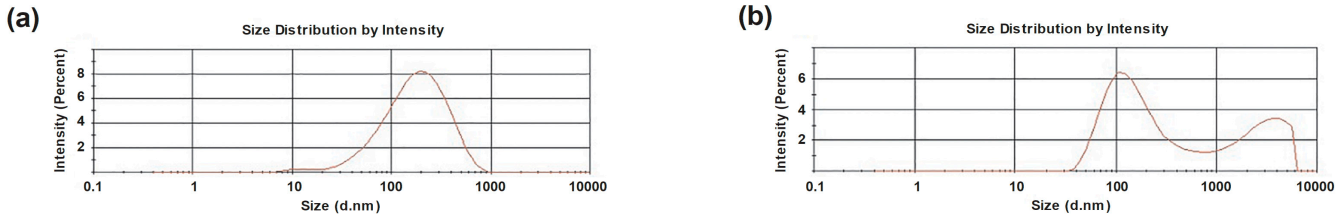

Figure S1. Particle size distribution of DA-CIT-SLNs-60 (a) and DA-CIT-SLNs-120 (b)

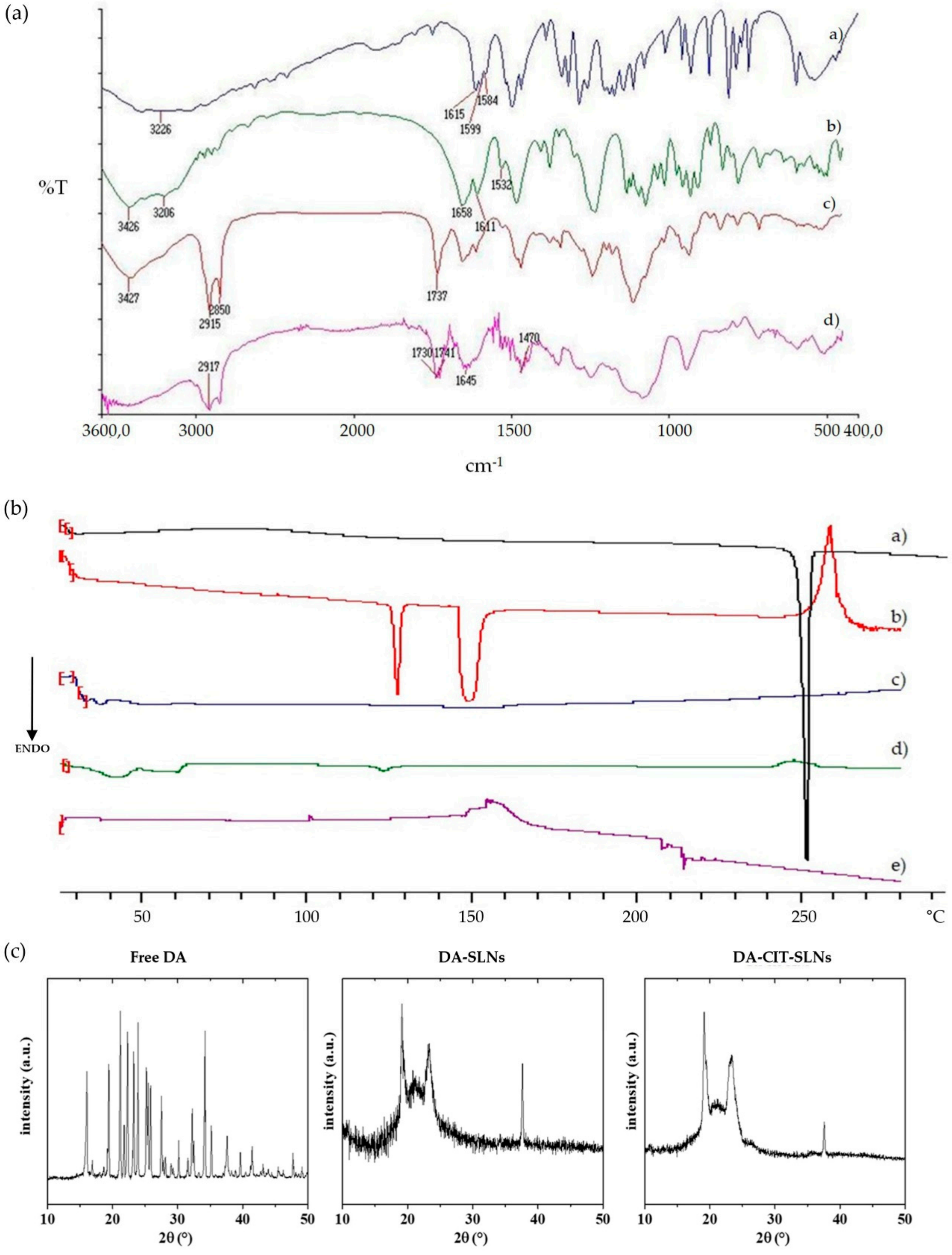
(a)
%T
a)
b)
c)
d)
3226
1615
1584
1599
3426
3206
1658
1532
1611
3427
2915
2850
1737
2917
1730
1741
1645
1470
3600,0
3000
2000
1500
1000
500 400,0
cm-1
(b)
a)
b)
c)
d)
e)
ENDO
50
100
150
200
250
°C
(c)
Free DA
DA-SLNs
DA-CIT-SLNs
intensity (a.u.)
2θ (°)
10
20
30
40
50

Figure S2: Panel **(a)**: FT-IR spectra of pure DA (a), pure CIT (b), DA-SLNs (c), DA-CIT-SLNs-60 (d). Panel **(b)**: DSC profiles of pure DA (a), pure CIT (b), DA-SLNs (c), CIT-SLNs (d), DA-CIT-SLNs-60 (e). Panel **(c)**: X-ray diffraction patterns of free DA (left); DA-SLNs (middle), DA-CIT-SLNs-60 (right). As for the FT-IR spectrum of CIT-SLNs and the X-ray diffraction patterns of free CIT and CIT-SLNs the reader is referred to Ref. [22].
